# Nervous Dyspepsia

**Published:** 1880-06

**Authors:** P. W. Van Peyma


### Translations.

### NERVOUS DYSPEPSIA.

FROM THE DUTCH BY P. W. VAN PEYMA, M. D.

Under this name W. O. Leube (*Archives fuer Klinische Medicin*, Dec. 1878), describes a pathological condition, which, although it gives rise to subjective symptoms similar to those observed in a number of other pathological conditions of the stomach, must yet be considered as a separate and distinct disease. The dyspeptic symptoms accompanying this disease are a feeling of fullness in the epigastrium, eructations, nausea, irregular appetite, tendency to cephalic congestion, weariness, sleepiness and headache. But in one particular there exists an important difference. In other diseases of the stomach, such as carcinoma ventriculi, gastric catarrh and gastric ulcer, accompanied by symptoms of dyspepsia, the digestion is disturbed and the food due to deficient peristaltic motion, remains in the stomach longer than usual. Leube finds this not to be the case in nervous dyspepsia. By means of a stomach pump and injections he satisfied himself that the stomach was empty within the normal time, seven hours after a meal. The water injected was returned clear and wanting in food.

In the differential diagnosis of nervous dyspepsia this fact obtains prominence. By this means we can exclude carcinoma ventriculi and chronic gastric catarrh. In the second place it is important with a view to diagnosis to note the negative result of a thoroughly applied dietetic regimen; this in the case of gastric catarrh being seldom without its effect. Certain cases of gastric ulcer, which run their course with few marked symptoms. unaccompanied with pain or vomiting and where the digestion is complete within the normal time, are most likely to give rise to a mistaken diagnosis.

In doubtful cases, both the uselessness of the thoroughly employed dietetic course, and the good results obtained from the employment of electricity over the region of the stomach during digestion, serve to render the existence of nervous dyspepsia probable. According to Leube, this disease is not rare and is generally observed in the higher classes within the first ten years after puberty. In accounting for the symptoms of nervous dyspepsia, Leube calls attention to what takes place physiologically after a somewhat hearty meal. The then occurring symptoms—the feeling of fullness in the epigastrium, the tendency to cephalic congestion, the disinclination to mental exertion, the weariness of the limbs, the sleepiness, can hardly be considered as the direct influence of the digestion upon the nervous system.

According to Leube, it is not so much the absorption of the products of chemical change, although this probably serves as a moderate stimulant to the nervous system, but later the purely mechanical irritation to which the gastric nerves are subject upon the introduction of food, and through which the whole nervous system is secondarily affected. In nervous dyspepsia the above nervous symptoms are observed in an increased degree, and this, because of the abnormal reaction of the gastric nerves to a normal stimulant, viz: the digestion of food. The cause of this abnormal reaction must be sought for in the gastric nerves themselves, although we may be unable, as in the case of the other so-called neurosis, to point out the anatomical changes. Some cases are incurable, and these hardly admit of relief. In the treatment of this obstinate disease, remedies tending to invigorate the general nervous system are particularly to be noted. The most important is the cold water cure. The use of iron and quinia internally, and electricity, both the induced and the constant current, the latter both externally and internally. The latter method of application, Leube has of late discarded, as affording no particularly beneficial results. The food must be easy of digestion. Sea bathing and sojourning in a mountainous region are useful to complete the cure.— *Weekblad von het Nederlandsch. Tydschraft voor Geereeskunde.*

